# Small-Sided Games vs. Running-Based High-Intensity Interval Training: An Exploratory Study of the Effects on Physical Performance and Internal Load in Under-11 Male Football Players

**DOI:** 10.3390/sports14030114

**Published:** 2026-03-13

**Authors:** Marco Panascì, Vittoria Ferrando, Carlo Castagna, Gennaro Apollaro, Piero Ruggeri, Emanuela Luisa Faelli

**Affiliations:** 1 Department of Experimental Medicine, Section of Human Physiology, University of Genoa, 16132 Genoa, Italy; marco.panasci@unige.it (M.P.); piero.ruggeri@unige.it (P.R.); emanuela.faelli@unige.it (E.L.F.); 2 Centro Polifunzionale di Scienze Motorie, University of Genoa, 16132 Genoa, Italy; gennaro.apollaro@edu.unige.it; 3 Department of Education and Sport Science, Pegaso Open University, 80143 Naples, Italy; castagnac@libero.it; 4 Department of Sports Science and Clinical Biomechanics, SDU Sport and Health Sciences Cluster (SHSC), University of Southern Denmark, 5230 Odense, Denmark; 5 Fitness Training and Biomechanics Laboratory, Technical Department, Italian Football Federation (FIGC), 50135 Coverciano, Italy; 6 Department of Neuroscience, Rehabilitation, Ophthalmology, Genetics and Maternal Child Health, University of Genoa, 16132 Genoa, Italy

**Keywords:** children, football, high-intensity interval training, performance, compliance

## Abstract

Background: This study aimed, in U11 male football players, (i) to investigate the effects of an 8-week running-based HIIT or SSGs program on aerobic fitness, neuromuscular performance and internal load, and (ii) to compare training-induced changes in performance variables between training modalities. Methods: Sixteen U11 football players were randomly assigned to either the SSGs group (4 vs. 4 format, 5 × 3 min with 1 min of rest between bouts) or the HIIT group (5 × 3 min of 15 s running at 100% peak velocity (Vpeak) alternating with 15 s of recovery, and 1 min of rest between sets). The intervention period lasted 8 weeks. Aerobic fitness (Yo-Yo Intermittent Recovery Level 1 Children’s Test, YYIR1C), sprint time performance (10 m and 20 m sprints tests) and change-of-direction (COD) ability (Arrowhead Agility Test) were assessed before and after the intervention. Heart rate (HR) and rating of perceived exertion (RPE) were assessed as indices of internal load. Results: Both SSGs and running-based HIIT produced comparable improvements in YYIR1C distance, Vpeak (*p* < 0.05), with no significant change in the between-group difference. Neuromuscular gains occurred only after SSGs (*p* < 0.05), with similar 10 m sprint improvements between groups but superior 20 m gains for SSGs (*p* < 0.01). COD ability improved in both groups (*p* < 0.05), with broader enhancements following SSGs (*p* < 0.05). Finally, running-based HIIT elicited greater HRpeak and higher RPE than SSGs (*p* < 0.05) across most intervention weeks. Conclusions: In U11 male football players, both SSGs and running-based HIIT effectively improved aerobic fitness and COD performance. However, SSGs may offer additional benefits for sprint development with lower perceived psychological stress.

## 1. Introduction

Youth football is characterized by a wide range of intermittent activities, including standing, walking, running at various intensities, jumping, tackling, shooting, and controlling the ball under pressure [1,2]. In young players, the development of these physical capacities is essential not only for match performance but also for long-term athletic development.

Childhood represents a critical phase of development, and it is considered a sensitive period during which appropriate training stimuli may induce substantial physiological and neuromuscular adaptations [3,4]. Compared with adolescents and adults, children exhibit distinct physiological characteristics, such as a lower anaerobic capacity, a reduced glycolytic activity, and greater reliance on oxidative metabolism during high-intensity exercise [5,6]. In addition, compared to their adult counterparts, likely due to metabolic and neuromuscular differences, children tend to recover more from high-intensity exercise, showing less fatigue and a quicker return to the next performance [7,8]. Understanding these age-related characteristics is crucial for the design of appropriate and effective training programs, as children may respond differently to high-intensity exercise compared with adolescent or adult athletes.

High-Intensity Interval Training (HIIT) is one of the most commonly used training strategies in football and is widely used to improve aerobic fitness and high-intensity running performance. A typical running-based High-Intensity Interval Training (HIIT) protocol consists of structured bouts of exercise performed at intensities close to maximal aerobic speed (MAS), defined as the minimal running velocity required to elicit maximal oxygen uptake, interspersed with short recovery periods. This approach allows for precise control of training intensity and work-to-rest ratios [9]. In addition, HIIT exercise intensity is typically quantified using internal load indicators such as mean and maximal heart rate (HRmean and HRmax) and rating of perceived exertion (RPE) [10].

Previous studies have demonstrated that this training modality effectively enhances aerobic performance and exercise tolerance in young football players [1,2,11].

An alternative HIIT-based approach frequently used in youth football is Small-Sided Games (SSGs). This training format involves modified game conditions with reduced pitch dimensions, fewer players, and specific rule constraints, enabling the execution of football-specific actions under intermittent high-intensity conditions [12]. SSGs are characterized by highly variable movement patterns, including frequent accelerations, decelerations, and changes of direction, potentially providing a comprehensive stimulus for both aerobic and neuromuscular development [13]. Moreover, SSGs allow the integration of physical conditioning with technical and tactical components, thereby increasing ecological validity in youth training environments [14].

Previous research reported that, in young football players, both running-based HIIT and SSGs induce comparable effects in key performance-related variables, including maximal oxygen uptake, running performance, and football-specific indicators such as total distance covered, number of sprints, and ball contacts [2]. However, the relative effectiveness of these two training approaches in prepubertal football players, particularly when compared to neuromuscular performance outcomes, remains unclear.

Despite the growing body of literature examining the effects of different HIIT formats in adult and adolescent football players, limited research has investigated the impact of running-based HIIT and SSGs on performance-related physical capacities such as sprint speed and change-of-direction ability in prepubertal players. These capacities, in fact, are highly relevant for football performance and may be especially responsive to training stimuli during childhood [15].

Therefore, the aim of the present study was, in U11 male football players, (i) to investigate the effects induced by an 8-week running-based HIIT or SSGs program on aerobic fitness, neuromuscular performance (sprint time and change-of-direction ability), as well as internal load; and (ii) to compare training-induced changes in performance variables between two training modalities.

It was hypothesized that both training modalities would be associated with significant improvements in aerobic fitness. Moreover, it was expected that the SSG format, thanks to its greater neuromuscular involvement and football-specific movement demands, might promote greater improvements in sprint and change-of-direction performance compared with running-based HIIT, thus potentially resulting in more enjoyable training.

## 2. Materials and Methods

### 2.1. Sample Size

An a priori sample size calculation was performed based on previously reported effects of HIIT on aerobic performance in young male football players [11,16]. The calculation was conducted using G*Power software (3.1.9.7 Heinrich-Heine-Universität Düsseldorf, Düsseldorf, Germany), assuming a two-tailed independent-samples *t*-test to detect between-group differences, with an alpha level of 0.05, a statistical power (1 − β) of 0.90, and an anticipated effect size (Cohen’s d) of 0.60. Based on these parameters, a minimum of 16 participants (8 per group) were required.

### 2.2. Participants

Sixteen prepubertal Under-11 football players were recruited from a youth Italian professional football academy. Participants’ anthropometric and training characteristics are reported in Table 1. Participants and their parents or legal guardians were informed about the purpose and procedures of the study and received detailed written information regarding the experimental protocol, potential risks or discomforts, and the right to withdraw from the study at any time without consequences. Then, written informed consent was obtained for all participants.

The study was conducted in accordance with the Declaration of Helsinki and approved by the local ethics committee of the university on 18 November 2025 (approval number: 2025/93).

### 2.3. Experimental Design

This parallel, matched-group design compared the effects of Small-Sided Games (SSGs) and running-based High-Intensity Interval Training (HIIT) on physical and neuromuscular performance as well as internal load in prepubertal football players. Before the commencement of the experimental protocol, to become accustomed to the training modalities and testing procedures, thereby improving measurement reliability, all participants completed a 2-week familiarization period. The study was conducted during the first half of the U11 season, approximately 8 weeks after the beginning of the season. The experimental protocol included pre-intervention testing, an 8-week training intervention, and post-intervention testing. Pre- and post-intervention assessments were conducted during the week immediately before and after the intervention period, respectively, and included (1) the Yo-Yo Intermittent Recovery Test Level 1 for Children (YYIR1C); (2) 10 m and 20 m sprint time tests; and (3) the Arrowhead Agility Test.

After pre-intervention assessments, participants were allocated to either the SSGs group (n = 8) or the HIIT group (n = 8) using a matched-pairs procedure based on aerobic fitness (YYIR1C) and neuromuscular performance (10 m and 20 m sprint tests and Arrowhead Agility Test) to ensure balanced groups. The intervention was conducted over the same training period for both groups and was integrated into the players’ regular weekly training schedule. Specifically, within the weekly training program, both groups performed the specific experimental training methods (SSGs or running-based HIIT) twice per week, replacing part of the conditioning component of their standard training sessions and continued to carry out two standardized technical–tactical training sessions per week (60 min) under the supervision of the same coaching staff, and they played matches on the weekends. Internal load was also monitored during sessions using heart rate (HR) and rating of perceived exertion (RPE) measures. All tests and training sessions were conducted on a grass field under similar environmental conditions and at the same time of day (15–16 °C, 55–60% relative humidity, and low wind: <2 m·s^−1^, 5:00 p.m. ± 1 h) to minimize potential circadian effects.

**Figure 1:**
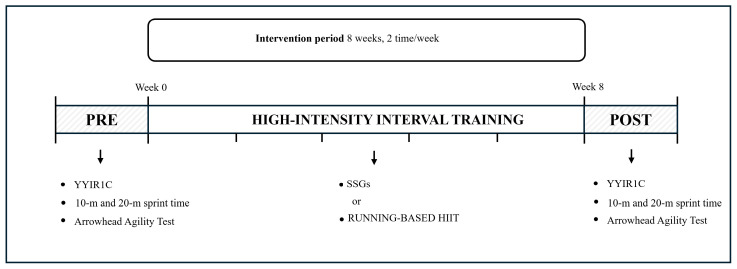
Experimental design. YYIR1C, Yo-Yo Intermittent Recovery Test Level 1 for Children; SSGs, Small-Sided Games; HIIT, High-Intensity Interval Training.

The experimental protocol is presented in Figure 1.

### 2.4. Testing Sessions

Each test was preceded by a standardized warm-up consisting of 10 min of self-paced running, 2 min of skipping, and 2 min of striding drills over 10 m and 30 m, respectively, and concluded by 2 min of passive recovery. Verbal encouragement was provided throughout all tests to ensure maximal effort. Participants wore football boots during all testing sessions.

Each participant performed one trial for each test, as all participants were familiar with the testing protocols. Aerobic fitness was assessed using the Yo-Yo Intermittent Recovery Test Level 1 for Children, while neuromuscular performance was evaluated using the 10 and 20 m Sprint tests and the Arrowhead Agility Test. Aerobic and neuromuscular performance assessments were conducted on two days separated by 48 h. All testing sessions followed the same test order. All test protocols were well tolerated, and participants completed each test without complications; no adverse events such as dizziness, light-headedness, or nausea were reported.

#### 2.4.1. Yo-Yo Intermittent Recovery Test Level 1 for Children (YYIR1C)

Yo-Yo Intermittent Recovery Test Level 1 for Children (YYIR1C) protocol involved 2 × 16 m repeated shuttle runs at progressive speeds, dictated by calibrated acoustic signals, and interspersed with 10 s of active recovery within a 4 m zone. Participants were instructed to complete as many shuttles as possible until volitional exhaustion. The test ended when the participant failed to reach the finishing line in accordance with the audio signal on two consecutive occasions. Total distance covered (TD, m) during the test was recorded as the primary outcome measure of aerobic fitness. In addition, during the YYIR1C, participants’ peak velocity (Vpeak) was determined.

#### 2.4.2. 10 m and 20 m Sprint Tests

The 10 m and 20 m sprints were measured using timing gates (Witty System; Microgate, Bolzano, Italy) positioned at a height of 0.70 m and perpendicular to the running direction. Participants performed three maximal 10 m sprints from a stationary start, with the front foot placed 0.5 m behind the starting line. Each sprint was separated by 2 min of passive recovery. Following a 10 min rest period, participants completed a specific sprint warm-up, which included three progressive 20 m submaximal build-up sprints, before performing three maximal 20 m sprint trials. Participants were instructed to run both the 10 m and 20 m sprints at maximal effort. For statistical analysis, the fastest time recorded among trials in both the 10 m and 20 m sprints was used.

#### 2.4.3. Arrowhead Agility Test

**Figure 2:**
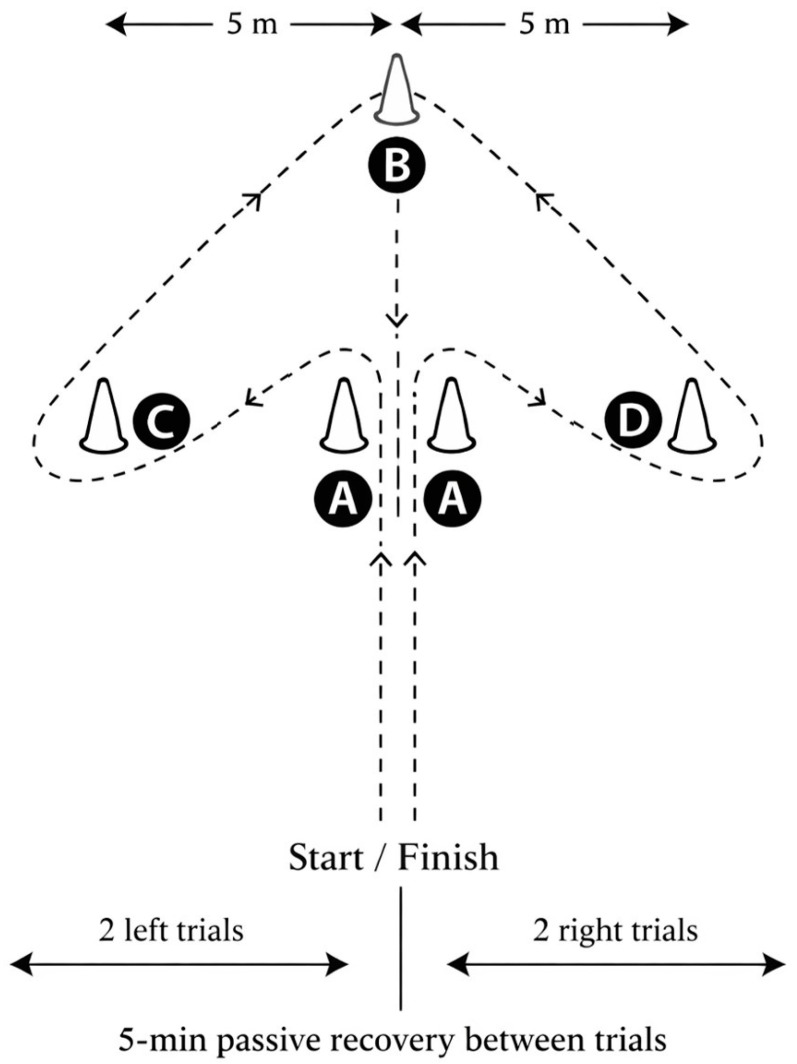
Schematic representation of the Arrowhead Agility Test. Participants sprint from the start zone to the middle marker (A), then turn left (C) or right (D), depending on the trial, to sprint around the side marker. They then sprint around the top marker (B) before returning through the finish zone. Dotted lines indicate movement paths, and arrows represent the running direction.

During the Arrowhead Agility Test (AAT; Figure 2), participants adopted a sprint starting position with both feet placed behind the starting line. Upon initiation, players sprinted forward to the central marker, changed direction to sprint through the lateral marker, continued toward the far marker, and finally returned through the start/finish line as quickly as possible. Each participant completed 4 maximal trials, consisting of 2 trials with changes in direction to the left and 2 to the right, with 5 min of passive recovery between trials. Performance times were recorded in seconds using timing gates (Witty System; Microgate, Bolzano, Italy) positioned at the start/finish line. The fastest time recorded for each direction (left and right) was retained for analysis.

Importantly, a recovery period of 10 min was included between completing the 10 m and 20 m sprint tests and the AAT to minimize fatigue and to ensure the optimal subsequent test performance.

### 2.5. Training Intervention

Participants were randomly assigned to either the Small-Sided Games (SSGs) group or the High-Intensity Interval Training (HIIT) group. Throughout the 8-week intervention period, both groups completed two experimental training sessions (SSGs or HIIT) per week plus two technical–tactical sessions/week. Therefore, participants completed a total of 16 experimental training sessions.

In all experimental sessions, heart rate (HR) and rating of perceived exertion (RPE) were recorded to quantify internal load.

During both SSGs and HIIT sessions, time spent above 90% of maximal heart rate (T@90%HRmax) was quantified to ensure comparability of the training stimulus. The average data obtained from the Monday and Thursday sessions indicated that T@90%HRmax was comparable between the two experimental training formats on both days (Monday: SSGs 6.71 ± 1.73 min vs. HIIT 7.75 ± 2.23 min, *p* = 0.16; Thursday: SSGs 7.24 ± 2.20 min vs. HIIT 8.08 ± 2.53 min, *p* = 0.33). Moreover, both experimental sessions lasted approximately 50 min.

Weekly training program description of both groups is reported in Table 2.

#### 2.5.1. Small-Sided Games (SSGs)

**Figure 3:**
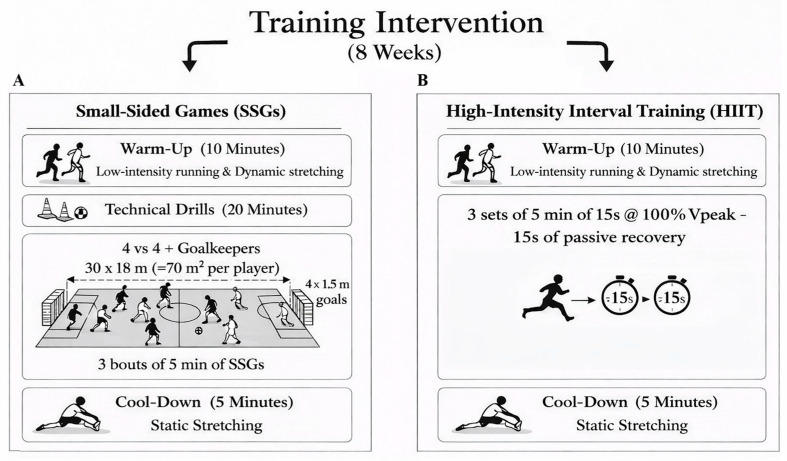
Description of both Small-Sided Games (SSGs) and High-Intensity Interval Training (HIIT) protocols. (**A**) Small-Sided Games; (**B**) High-Intensity Interval Training.

The SSGs format consisted of 4 vs. 4 with two goalkeepers, continuous coach verbal encouragement and no touch limit to maintain high intensity throughout each bout. The pitch measured 30 × 18 m (≈70 m^2^ per player) and included two 4 × 1.5 m goals (Figure 3A). Each SSG session started with a 10 min standardized warm-up, including low-intensity running and dynamic stretching exercises, followed by 20 min of technical drills. Then, participants performed 3 bouts of 5 min each separated by 1 min of passive recovery. The session concluded with 5 min of cool-down, including static stretching exercises.

During SSGs, official football rules were applied, except for the offside rule. Furthermore, the time lost due to major injury stoppages was excluded from the total training duration to preserve the training objectives, maximizing ball-in-play time and simulating match-specific scenarios. In all SSG sessions, the internal load was also monitored.

A description of the SSG protocol is reported in Figure 3A.

#### 2.5.2. Running-Based High-Intensity Interval Training (HIIT)

The HIIT group performed three sets of 5 min running intervals consisting of linear running bouts of 15 s of runs at 100%Vpeak alternating with 15 s of passive recovery, with 1 min of rest between sets (Figure 3B). Speed intensities were individualized based on each player’s Vpeak, determined during the baseline Yo-Yo Intermittent Recovery Test Level 1 for Children (YYIR1C). Each HIIT session started with a 10 min standardized warm-up, including low-intensity running and dynamic stretching exercises, followed by 20 min of technical drills and concluded with a 5 min cool-down involving static stretching exercises. The internal load was measured throughout the training sessions. A description of the High-Intensity Interval Training protocol is reported in Figure 3B.

### 2.6. Outcome Measures

#### 2.6.1. Aerobic Fitness

Aerobic fitness was assessed using the Yo-Yo Intermittent Recovery Test Level 1 for Children (YYIR1C), as it is considered a field-based test with established validity and reliability in pediatric athletic populations [17]. Total distance covered during the test (YYIR1C distance) was chosen as an index of aerobic fitness.

#### 2.6.2. Neuromuscular Performance

Sprint performance was evaluated through 10 m and 20 m sprint tests, which are widely recommended for the assessment of linear speed in academy football players, following the protocol outlined by Moran and colleagues (2020) [18].

Change-of-direction (COD) ability was assessed using the Arrowhead Agility Test (AAT), a football-specific field test commonly used to evaluate multidirectional sprinting performance [19]. The AAT has demonstrated good intersession reliability (ICC = 0.80–0.83; CV = 1.25–2.21%) and practical utility for profiling COD capacity in football players [19]. Performance times recorded during both the 10 m and 20 m sprint tests and AAT were chosen as indices of neuromuscular performance.

#### 2.6.3. Internal Load Monitoring

Internal load was quantified using heart rate (HR) and rating of perceived exertion (RPE). HR monitoring is a well-established method for assessing internal load in youth soccer players and correlates strongly with both external load measures and perceived exertion [20]. Heart rate was continuously recorded at 5 s intervals using a chest strap monitor (Polar H10, Polar Electro OY, Kempele, Finland). HR values were monitored throughout all training bouts. Mean heart rate (HRmean) and peak heart rate (HRpeak) were expressed as percentages of the individual maximal HR achieved in the YYIR1C (%HRmax) and defined as the average and highest values, respectively, recorded during each training session.

Perceived exertion was assessed using the Borg Category-Ratio 10 (CR-10) scale. This validated scale ranges from 0 (no effort) to 10 (maximal effort), and it has been widely used to monitor internal load in soccer and other team sports, reliably reflecting training intensity in young athletes [21,22]. RPE value was assessed 10 min after the end of each training session.

Prior to the intervention, participants were familiarized with the scale and anchoring procedures to ensure accurate and consistent reporting.

### 2.7. Statistical Analysis

Data were analyzed using IBM SPSS Statistics for Windows, version 25.0 (IBM Corp., Armonk, NY, USA). The Shapiro–Wilk test revealed the nonnormal distribution of the considered variables. Therefore, descriptive data are presented as median (interquartile range) [minimum–maximum]. Although the a priori calculation of the sample size was based on parametric assumptions, non-parametric tests were applied since normality assumptions were not satisfied.

Within-group differences between pre- (PRE) and post-training (POST) assessments for each performance parameter (i.e., YYIR1C, 10 m and 20 m sprints time, and Arrowhead Agility Test) were analyzed using the Wilcoxon Signed-Rank test. Between-group differences for the delta (Δ) values (POST–PRE) of performance parameters, as well as physiological and perceptual measures (i.e., HR and RPE) recorded during each week of training, were assessed using the Mann–Whitney U test.

The standardized median difference (SMD) was used as the effect size and calculated using the formula SMD = median_A_ − median_B_/IQR_pooled_. According to Cohen [23], SMD values of 0.00 to 0.50 indicate small effects; 0.51–0.79, medium effects; and ≥0.80, large effects. Statistical significance was accepted at *p* < 0.05.

## 3. Results

### 3.1. Aerobic and Neuromuscular Performance in the SSGs Group

In the SSGs group, significant improvements in both aerobic and neuromuscular performance were observed.

As regards aerobic fitness, participants showed a significant increase in YYIR1C distance (Z = −2.524; *p* = 0.012; SMD = 2.15, large), as well as in peak velocity (Vpeak) (Z = −2.379; *p* = 0.017; SMD = 2.00, large).

Concerning neuromuscular performance, values significantly improved in both the 10 m (Z = −2.103; *p* = 0.035; SMD = 0.43, small) and 20 m sprint time (Z = −2.521; *p* = 0.012; SMD = 1.14, large). Similarly, COD ability significantly improved for both the right (Z = −2.521; *p* = 0.012; SMD = 2.42, large) and left directions (Z = −2.521; *p* = 0.012; SMD = 1.12, large). The results of the statistical analysis are reported in Table 3.

### 3.2. Aerobic and Neuromuscular Performance in the HIIT Group

The HIIT group showed significant improvements in both aerobic and neuromuscular performance, although the magnitude and statistical significance varied across tests.

For aerobic fitness, participants demonstrated a significant increase in YYIR1C distance (Z = −2.521; *p* = 0.012; SMD = 1.20, large) and Vpeak (Z = −2.414; *p* = 0.016; SMD = 1.00, large).

As regards neuromuscular performance, sprint performance showed no significant improvements in both the 10 m (Z = −1.823; *p* = 0.068; SMD = 0.42, small) and 20 m sprint time (Z = −1.414; *p* = 0.157; SMD = 0.42, small). While COD ability significantly improved for both left (Z = −2.533; *p* = 0.011; SMD = 0.40, small) and right sides (Z = 1.960; *p* = 0.048; SMD = 0.66, medium). The results of the statistical analysis are reported in Table 4.

### 3.3. Between-Group Comparisons (Δ Values)

**Figure 4:**
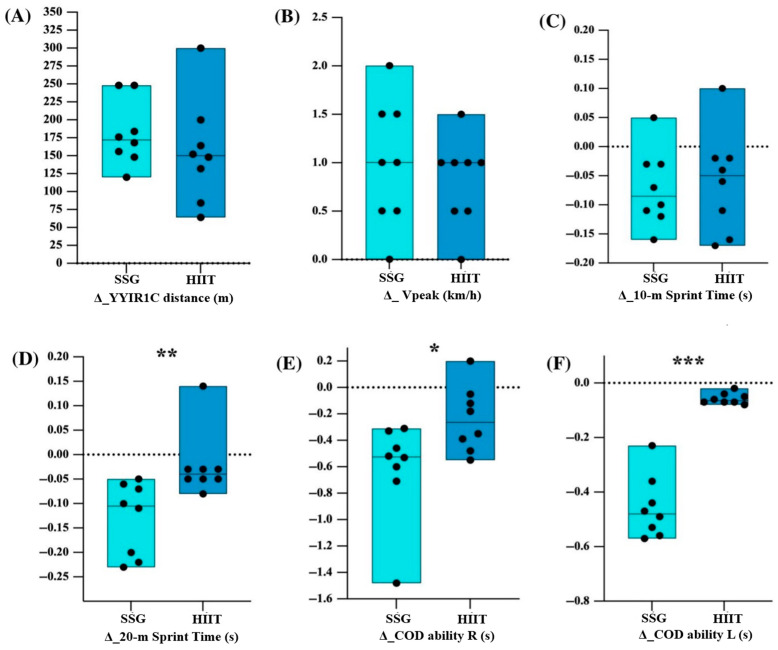
Between-group comparisons of changes (Δ) in performance variables in SSGs and HIIT groups. Panels show changes in (**A**) YYIR1C distance, (**B**) Vpeak, (**C**) 10 m sprint time, (**D**) 20 m sprint time, (**E**) COD ability right side and (**F**) COD ability left side. YYIR1C, Yo-Yo Intermittent Recovery Test Level 1 for Children; SSGs, Small-Sided Games; HIIT, High-Intensity Interval Training; Vpeak, peak velocity; COD, change of direction; R, right; L, left. * *p* ≤ 0.05, ** *p* ≤ 0.01, *** *p* ≤ 0.001.

Between-group comparisons of the changes (Δ) in both aerobic and neuromuscular performance parameters are reported in Figure 4.

Specifically, for the aerobic fitness, the SSGs group increased the YYIR1C distance (SSGs: 20%; HIIT: 17%) and Vpeak (SSGs: 7%; HIIT: 6%) showed no significant between-group differences (Δ_YYIR1C: U = 21.50; *p* = 0.269; SMD = 0.25, small; Δ_Vpeak: U = 26.50; *p* = 0.548; SMD = 0.001, small) (Figure 4A,B).

As regards neuromuscular performance, both groups showed similar 10 m sprint time improvements with no significant differences between groups (SSGs: −3%; HIIT: −3%; U = 29.00; *p* = 0.752; SMD = 0.36, small) (Figure 4C), while the 20 m sprint time significantly improved in the SSGs group than in the HIIT group (SSGs: −4%; HIIT: −1%) (U = 4.50; *p* = 0.004; SMD = 0.61, medium) (Figure 4D). Moreover, the COD ability of the SSGs group obtained a significant improvement in both directions compared to the HIIT group. Right-side COD performance improved by 7% in SSGs versus 3% in HIIT groups (U = 12.00; *p* = 0.036; SMD = 0.73, medium) (Figure 4E) and left-side COD performance improved by 5% versus 1% (U = 0.00; *p* = 0.001; SMD = 3.36, large) (Figure 4F).

The results of the between-group statistical comparisons are reported in Table 5.

### 3.4. Internal Load Monitoring

Weekly internal load responses, assessed through HRmean (as %HRmax), HRpeak (as %HRmax), and rating of perceived exertion (RPE), are reported in Table 6 (absolute HRmean and HRpeak values are shown in Supplementary Table S1). No statistically significant between-group differences were observed for HRmean across individual weeks (e.g., Week 1: U = 30.50, *p* = 0.916, SMD = 0.001, small; Week 2: U = 24.00, *p* = 0.400, SMD = 0.001, small). When considering the cumulative HRmean over the 8-week intervention, a medium effect size was observed (U = 14.00, *p* = 0.065, SMD = 0.50), indicating a trend toward higher mean cardiovascular load in one training modality, without reaching statistical significance.

In contrast, the HIIT group demonstrated a significant difference in HRpeak over several weeks, with medium to large effect sizes observed during Week 2 (U = 9.50, *p* = 0.021, SMD = 0.78), Week 3 (U = 5.00, *p* = 0.003, SMD = 1.90), and Week 4 (U = 7.00, *p* = 0.010, SMD = 0.63) compared to the SSGS group. Moreover, the total HRpeak across the intervention period significantly differed between groups (U = 3.00, *p* = 0.001, SMD = 0.63, large), suggesting greater maximal cardiovascular responses associated with the HIIT group compared to the SSGs group.

RPE values were consistently higher in the HIIT group throughout the intervention, with significant between-group differences observed in most weeks (e.g., Week 1: U = 8.00, *p* = 0.010, SMD = 0.63, medium; Week 2: U = 6.50, *p* = 0.005, SMD = 1.00, large). The cumulative RPE over the 8-week period also revealed a large effect size (U = 1.00, *p* < 0.001, SMD = 1.41), indicating that the SSGs group reported significantly lower RPE values compared with the HIIT group. The results of the statistical analysis of internal load variables are reported in Table 6.

## 4. Discussion

The present study investigated in under-11 male football players, the effects of two different HIIT modalities, such as Small-Sided Games (SSGs) and running-based HIIT, on aerobic and neuromuscular performance as well as internal load in over an eight-week intervention period.

Our findings indicated that both SSGs and HIIT induced similar improvements in aerobic fitness parameters, such as YYIR1C distance and Vpeak, with no significant between-group differences in the magnitude of change.

As regards the neuromuscular performance, only the SSGs showed a significant improvement both in the 10 m and 20 m sprint tests. However, between-group comparison of changes indicated that initial acceleration adaptations (10 m sprint) were similar, while SSGs’ superiority appears distance-dependent (20 m only). 

In addition, both SSGs and running-based HIIT induced similar improvements in change-of-direction (COD) ability in both directions. In this sense, the SSGs induced a broader improvement in both directions compared to the running-based HIIT format. 

Finally, HIIT format consistently elicited higher internal load responses, as indicated by greater HRpeak values and higher RPE scores across most weeks of the intervention period.

### 4.1. Effects on Aerobic Performance

Investigating the effects of different HIIT modalities plays a fundamental role in designing appropriate training protocols in U11 male football players. Previous evidence, conducted in young football players, indicated that running-based HIIT is a training modality specifically designed to maximize time spent near maximal oxygen uptake [10], whereas SSGs may induce similar cardiovascular stimuli through continuous player involvement, reduced numbers of participants, and limited recovery phases [24].

The present study showed that, in prepubertal male football players, both SSGs and running-based HIIT formats were effective in improving aerobic fitness, as reflected by increases in YYIR1C distance and peak running velocity. Our results are in line with the previous literature that reported, in young male football players, an improved aerobic performance following both these HIIT modalities [11,13,16]. It is plausible that the intermittent high-intensity nature of both formats provided sufficient cardiovascular stimulus to promote aerobic fitness adaptations. However, as no direct physiological measures were collected in our study, any metabolic adaptations should be interpreted with caution.

### 4.2. Effects on Neuromuscular Performance

As regards neuromuscular performance, sprint times on 10 m and 20 m, as well as COD ability, were assessed.

Sprint time performance (10 m and 20 m sprint) significantly improved only in the SSGs, while the running-based HIIT regime did not show any significant change after the intervention period. However, the analysis of median changes (Δ) on 10 m sprint performance revealed a similar percentage of performance increment between the two training modalities, with sprint time reduced by 3% following the respective interventions. These results suggest that both formats are able to induce similar initial acceleration adaptations. This is in line with previous evidence indicating that short sprint performance may be less sensitive to training modality when considering only the initial acceleration phase [25].

In contrast, for the 20 m sprint, the SSGs regime demonstrated a broader significative percentage of improvement compared to the running-based HIIT mode (−4% vs. −1%). The medium effect size associated with this difference highlights the superiority of SSGs in enhancing sprint performance over longer acceleration distances (20 m), thus indicating that the SSGs regime can induce more substantial adaptations in sprint capacity. In line with the results of this present study, a meta-analytical comparison by Clemente et al. [15] reported greater improvements in sprint performance following SSGs compared to running-based interventions. Although SSGs may provide a more specific stimulus for enhancing sprint performance over longer acceleration distances, the underlying neuromuscular mechanisms remain speculative, as no direct physiological measures or external load were collected.

The change-of-direction (COD) ability in both right and left directions, assessed using the Arrowhead Agility Test, significantly improved following both training modalities, thus indicating that both SSGs and running-based HIIT are effective in inducing multidirectional movement performance improvements. However, the between-group comparison highlighted a broader significative percentage of improvement in SSGs compared to running-based HIIT, showing COD right-side improvements of 7% versus 3% and COD left-side improvements of 5% versus 1%. These findings suggest the potential influence of training specificity, as SSGs involve frequent high-intensity accelerations, decelerations, and rapid changes in movement direction under unpredictable conditions, which closely resemble standardized COD testing [15,26].

The bilateral improvements observed further suggest a generalized enhancement in agility rather than direction-specific effects. In contrast, while running-based HIIT improved absolute COD performance, it lacks the multidirectional and perceptual components inherent to SSGs, which may explain the smaller relative changes.

However, given the short duration of the intervention, the observed improvements in COD performance could be due to repeated exposure to high-intensity accelerations, decelerations, and directional changes inherent to this training modality, as well as the potential contribution of motor learning or familiarization effects.

Overall, these results support the principal effects of training specificity of the SSGs modality and are in line with previous evidence reporting the effectiveness of SSGs in enhancing COD ability in young male football players [15,27]. Indeed, the multidirectional nature of SSGs imposes repeated braking, re-acceleration, and rapid changes in movement direction in response to dynamic environmental constraints. These demands closely resemble those assessed in standardized change-of-direction tests such as the Arrowhead Agility Test, which may explain the superior transfer of SSGs training effects compared with running-based HIIT.

### 4.3. Internal Load Monitoring

The assessment of internal load, derived from heart rate mean (HRmean), heart rate peak (HRpeak) and rating of perceived exertion (RPE), showed distinct responses between the SSGs and running-based HIIT modalities. Specifically, the running-based HIIT format consistently elicited higher internal load responses, as indicated by greater HRpeak values and higher RPE scores across most weeks of the intervention period. These findings are consistent with previous studies reporting that running-based HIIT format imposes greater internal load stress compared with SSGs [28,29]. In addition, several studies demonstrated that both RPE and HRpeak are usually lower during SSG sessions than in running-based HIIT, likely due to the intermittent nature of SSGs, the presence of a rest/recovery period and self-paced intensity [13,30].

Conversely, HRmean did not differ between the two training modalities throughout the intervention period, thus suggesting that both training modalities elicited a comparable overall cardiovascular demand despite differences in peak heart rate responses and perceived exertion.

Overall, our findings demonstrated that the SSGs regime is able to induce a lower subjective perception of effort, thus enhancing individual compliance and adherence more than the HIIT modality.

### 4.4. Limitations and Future Directions

This exploratory study has several limitations that should be acknowledged. First, the relatively small sample size may have increased the risk of type II errors and inflated effect size estimates, thereby reducing statistical power and limiting the generalizability of the findings beyond academy-level Italian under-11 players.

Second, external load parameters such as total distance covered, as well as the absence of direct physiological measurements, were not monitored, precluding verification of true comparability between training stimuli and limiting insight into the mechanical demands imposed.

Third, injury surveillance was not conducted, restricting the interpretation of potential adverse effects or injury-related risks associated with the training protocols.

Finally, the prepubertal status of participants was assumed based on their chronological age, as no biological maturation assessment (e.g., maturity offset, Tanner stage or peak height velocity) was performed. Given the established influence of maturation on sprint and change-of-direction development, this limitation reduces the interpretability of the neuromuscular outcomes. Inter-individual variability in maturation may also have contributed to the observed adaptations.

Future research should incorporate objective assessments of biological maturation and external load monitoring (e.g., GPS-based systems) to better account for developmental variability and to more accurately quantify mechanical load and neuromuscular specificity.

### 4.5. Practical Applications

From a practical perspective, in U11 football players, coaches and strength and conditioning professionals may consider implementing SSG sessions within the weekly training program to enhance aerobic and neuromuscular performance while maintaining a low perception of effort, which may support motivation, engagement, and long-term adherence.

In parallel, running-based HIIT sessions may represent a valid alternative modality for inducing aerobic fitness and change-of-direction ability improvements when the primary goal is to elicit high and tightly controlled cardiovascular stress.

## 5. Conclusions

The present study suggests that, in U11 male football players, both SSGs and running-based HIIT formats are effective for enhancing aerobic fitness and change-of-direction performance. However, SSGs may provide an additional stimulus for the development of sprint performance with a lower perceived psychological stress. Overall, given the small sample, our findings should be interpreted with caution and be limited to the academic level investigated.

## Figures and Tables

**Table 1:** Participant characteristics and results of YYIR1, 10 m and 20 m sprint time and Arrowhead Agility Test at the baseline.

	SSGs	HIIT	*p*-Values
Age (years)	10.50 ± 0.53	10.63 ± 0.52	n.s.
Height (m)	1.45 ± 0.06	1.47 ± 0.10	n.s.
Body mass (kg)	38.08 ± 7.16	37.43 ± 7.20	n.s.
BMI (kg/m^2^)	18.02 ± 2.84	17.30 ± 1.36	n.s.
TD YYIR1C (m)	898 ± 90	906 ± 82	n.s.
Vpeak (km/h)	14.25 ± 0.39	14.36 ± 0.38	n.s.
10 m sprint time (s)	2.03 ± 0.12	2.06 ± 0.10	n.s.
20 m sprint time (s)	3.53 ± 0.08	3.56 ± 0.08	n.s.
COD ability R (s)	9.05 ± 0.27	9.00 ± 0.27	n.s.
COD ability L (s)	9.14 ± 0.29	9.00 ± 0.21	n.s.

HIIT, High-Intensity Interval Training; SSGs, Small-Sided Games; BMI, Body Mass Index; YYIR1C, Yo-Yo Intermittent Recovery Test Level 1 for Children; TD, total distance; Vpeak, peak velocity; COD, change of direction; R, right; L, left; n.s., non significant.

**Table 2:** Weekly training program description.

Group	Monday	Tuesday	Thursday	Friday	Sunday
SSGs	20 min of technical drills 3 bouts of 5 min of SSGs	60 min of technical– tactical training	20 min of technical drills 3 bouts of 5 min of SSGs	60 min of technical– tactical training	Match
HIIT	20 min of technical drills 3 sets of 5 min of 15s@100%Vpeak-15s of passive recovery	60 min of technical– tactical training	20 min of technical drills 3 sets of 5 min of 15s@100%Vpeak-15s of passive recovery	60 min of technical– tactical training	

SSGs, Small-Sided Games; HIIT, High-Intensity Interval Training; Vpeak, peak of velocity.

**Table 3:** Changes from pre- to post-tests in SSGs group.

	SSGs	
	PRE Median (1stQ–3rdQ) (Min–Max)	POST Median (1stQ–3rdQ) (Min–Max)
YYIR1C distance (m)	882 (832–932) (812–1080)	1080 (1026–1109) (932–1248) *
Vpeak (km/h)	14.0 (14.0–14.5) (14.0–15.0)	15.0 (15.0–15.5) (15.0–16.0) *
10 m Sprint Time (s)	2.03 (1.92–2.14) (1.84–2.16)	1.96 (1.94–2.00) (1.81–2.06) *
20 m Sprint Time (s)	3.51 (3.48–3.61) (3.41–3.64)	3.40 (3.38–3.42) (3.35–3.47) *
COD Ability R (s)	9.19 (9.07–9.31) (8.78–9.50)	8.61 (8.47–8.71) (8.02–8.87) *
COD Ability L (s)	9.12 (8.98–9.34) (8.73–9.89)	8.65 (8.50–8.97) (8.29–9.33) *

YYIR1C, Yo-Yo Intermittent Recovery Test Level 1 for Children; SSGs, Small-Sided Games; Vpeak, peak velocity; COD, change of direction; R, right; L, left. * *p* ≤ 0.05.

**Table 4:** Changes from pre- to post-tests in HIIT group.

	HIIT	
	PRE Median (1stQ–3rdQ) (Min–Max)	POST Median (1stQ–3rdQ) (Min–Max)
YYIR1C Distance (m)	898 (836–960) (816–1048)	1064 (976–1128) (932–1212) *
Vpeak (km/h)	14.5 (14.0–14.5) (14.0–15.0)	15.0 (15.0–15.5) (15.0–15.5) *
10 m Sprint Time (s)	2.06 (1.97–2.15) (1.95–2.18)	2.00 (1.96–2.05) (1.89–2.12)
20 m Sprint Time (s)	3.59 (3.52–3.62) (3.37–3.62)	3.55 (3.50–3.59) (3.32–3.67)
COD Ability R (s)	9.04 (8.73–9.08) (8.51–9.22)	8.70 (8.34–8.98) (8.29–9.26) *
COD Ability L (s)	9.05 (8.93–9.11) (8.54–9.26)	8.98 (8.87–9.04) (8.52–9.22) *

YYIR1C, Yo-Yo Intermittent Recovery Test Level 1 for Children; HIIT, High-Intensity Interval Training; Vpeak, peak velocity; COD, change of direction; R, right; L, left. * *p* ≤ 0.05.

**Table 5:** Between-group comparisons of median changes (Δ) in performance variables in SSGs and HIIT groups.

	SSGs Median (95% CI) (1stQ–3rdQ) (Min–Max)	HIIT Median (95% CI) (1stQ–3rdQ) (Min–Max)
Δ_YYIR1C Distance (m)	172 (143–219) (150–232) (120–248)	150 (95–216) (96–191) (64–300)
Δ_Vpeak (km/h)	1.00 (0.45–1.55) (0.50–1.50) (0.00–2.00)	1.00 (0.43–1.20) (0.50–1.00) (0.00–1.50)
Δ_10 m Sprint Time (s)	−0.09 (−0.13–−0.02) (−0.12–−0.03) (−0.16–0.05)	−0.05 (−0.13–0.01) (−0.15–−0.02) (−0.17–0.10)
Δ_20 m Sprint Time (s)	−0.11 (−0.19–−0.07) (−0.22–−0.06) (−0.23–−0.05)	−0.04 (−0.08–0.03) (−0.05–−0.03) (−0.08–0.14) **
Δ_COD Ability R (s)	−0.53 (−0.93–−0.31) (−0.68–−0.36) (−1.48–−0.31)	−0.27 (−0.45–−0.03) (−0.46–−0.07) (−0.55–0.20) *
Δ_COD Ability L (s)	−0.48 (−0.55–−0.36) (−0.55–−0.38) (−0.57–−0.23)	−0.07 (−0.07–−0.04) (−0.07–−0.04) (−0.08–−0.02) ***

YYIR1C, Yo-Yo Intermittent Recovery Test Level 1 for Children; SSGs, Small-Sided Games; HIIT, High-Intensity Interval Training; Vpeak, Peak Velocity; COD, change of direction; R, right; L, left. * *p* ≤ 0.05, ** *p* ≤ 0.01, *** *p* ≤ 0.001.

**Table 6:** Internal load responses to SSGs and HIIT groups across an 8-week training period.

		SSGs Median (1stQ–3rdQ) (Min–Max)	HIIT Median (1stQ–3rdQ) (Min–Max)
HRmean (%HRmax)	Week 1	78 (76–79) (74–81)	78 (77–78) (76–79)
	Week 2	79 (77–80) (76–82)	79 (79–79) (79–79)
	Week 3	79 (77–80) (76–81)	80 (79–81) (78–82)
	Week 4	78 (78–79) (76–81)	80 (80–80) (77–81)
	Week 5	79 (78–80) (76–82)	81 (80–81) (78–83)
	Week 6	79 (79–80) (77–81)	80 (80–80) (79–83)
	Week 7	78 (77–81) (75–84)	80 (79–81) (77–82)
	Week 8	79 (79–80) (77–81)	80 (79–80) (78–82)
	Total	79 (78–79) (76–80)	79 (79–80) (79–81)
HRpeak (%HRmax)	Week 1	95 (95–96) (92–98)	96 (96–97) (94–100)
	Week 2	94 (93–95) (92–96)	96 (95–98) (94–99) *
	Week 3	94 (93–95) (91–96)	97 (96–97) (95–97) **
	Week 4	95 (94–95) (94–96)	95 (95–97) (95–100) **
	Week 5	95 (93–96) (92–98)	95 (95–97) (94–99)
	Week 6	95 (95–96) (95–97)	97 (96–98) (93–99)
	Week 7	95 (94–95) (94–97)	95 (95–96) (94–97)
	Week 8	95 (95–96) (91–98)	96 (95–97) (94–97)
	Total	95 (95–95) (93–96)	96 (95–97) (95–97) ***
RPE	Week 1	4 (3–4) (3–5)	5 (4–6) (4–6) **
	Week 2	4 (3–4) (3–4)	5 (5–6) (3–6) **
	Week 3	3 (3–4) (2–5)	5 (4–6) (4–6) **
	Week 4	4 (3–4) (3–5)	5 (4–6) (4–6) *
	Week 5	4 (3–5) (3–5)	6 (5–6) (5–7) **
	Week 6	4 (4–5) (3–5)	5 (5–6) (4–6) *
	Week 7	4 (3–5) (3–5)	5 (4–6) (3–6)
	Week 8	4 (3–4) (3–5)	5 (4–5) (4–6) **
	Total	4 (4–4) (4–4)	5 (5–5) (4–6) ***

HRmean, mean heart rate; HRpeak, peak heart rate; RPE, rating of perceived exertion. * *p* ≤ 0.05, ** *p* ≤ 0.01, *** *p* ≤ 0.001.

## Data Availability

Data generated or analyzed during this study are available from the corresponding author upon reasonable request.

## Supplementary Materials

The following supporting information can be downloaded at: https://www.mdpi.com/article/10.3390/sports14030114/s1, Table S1: HRmean and HRpeak responses to SSGs and HIIT groups across an 8-Week Training Period.
