# Supplementary material for: Small-Sided Games vs. Running-Based High-Intensity Interval Training: An Exploratory Study of the Effects on Physical Performance and Internal Load in Under-11 Male Football Players
Source: Sports (Basel). 2026 Mar 13;14(3):114. doi: 10.3390/sports14030114 (PMC13030078; doi:10.3390/sports14030114)
Supplement: Supplementary file 1 [file sports-14-00114-s001.zip › sports-4155982-supplementary.pdf]

**Supplementary Table S1.** HRmean and HRpeak responses to SSGs and HIIT groups across an 8-Week Training Period.

| | | SSGs<br>Median ($1^{st}$Q–$3^{rd}$Q) [Min–Max] | HIIT<br>Median ($1^{st}$Q–$3^{rd}$Q) [Min–Max] |
|---|---|---|---|
| **HRmean** | Week 1 | 160 (154–164)[153–167] | 163 (158–165)[158–166] |
| | Week 2 | 162 (158–166)[158–169] | 166 (164–167)[155–170] |
| | Week 3 | 163 (159–165)[158–165] | 165 (162–167)[161–168] |
| | Week 4 | 162 (159–164)[153–165] | 163 (162–165)[160–169] |
| | Week 5 | 162 (160–166)[158–168] | 165 (163–168)[162–169] |
| | Week 6 | 162 (161–164)[160–166] | 165 (162–168)[161–169] |
| | Week 7 | 161 (155–170)[153–173] | 164 (161–167)[161–168] |
| | Week 8 | 162 (161–165)[160–166] | 163 (162–166)[162–166] |
| | Total | 161 (160–164)[159–166] | 164 (163–165)[163–166] |
| **HRpeak** | Week 1 | 196 (164–168)[191–199] | 197 (196–199)[195–202] |
| | Week 2 | 192 (191–196)[190–198] | 198(194–201)[193–202] |
| | Week 3 | 193 (191–195)[191–196] | 197 (195–198)[194–201] |
| | Week 4 | 195 (192–197)[191–197] | 197 (195–201)[193–203] |
| | Week 5 | 195 (192–199)[192–199] | 198 (195–200)[190–203] |
| | Week 6 | 194 (191–198)[190–199] | 198 (194–202)[192–202] |
| | Week 7 | 195 (192–196)[190–196] | 196 (193–198)[190–199] |
| | Week 8 | 197 (192–197)[191–198] | 196 (195–198)[195–198] |
| | Total | 194 (194–195)[193–196] | 197 (196–198)[195–198] |
